# FSHB Genotype Identified as a Relevant Diagnostic Parameter Revealed by Cluster Analysis of Men With Idiopathic Infertility

**DOI:** 10.3389/fendo.2021.780403

**Published:** 2021-12-21

**Authors:** Henrike Krenz, Andrea Sansone, Sabine Kliesch, Joerg Gromoll, Maria Schubert

**Affiliations:** ^1^ Institute of Medical Informatics, University of Münster, Münster, Germany; ^2^ Department of Systems Medicine, Chair of Endocrinology and Medical Sexology, University of Rome Tor Vergata, Rome, Italy; ^3^ Department of Clinical and Surgical Andrology, Centre of Reproductive Medicine and Andrology (CeRA), University of Münster, Münster, Germany; ^4^ Institute of Reproductive and Regenerative Biology, Centre of Reproductive Medicine and Andrology (CeRA), University of Münster, Münster, Germany

**Keywords:** cluster, idiopathic male infertility, FSHB c.-211 G>T polymorphism, FSH (follicle stimulating hormone), segregation marker

## Abstract

**Introduction and Objectives:**

About 30-75% of infertile men are diagnosed with idiopathic infertility, thereby lacking major causative factors to explain their impaired fertility status. In this study, we used a large cohort of idiopathic infertile men to determine whether subgroups could be identified by an unbiased clustering approach and whether underlying etiologic factors could be delineated.

**Patients and Methods:**

From our in-house database Androbase^®^, we retrospectively selected patients (from 2008 to 2018) with idiopathic male infertility (azoo- to normozoospermia) who fit the following selection criteria: FSH ≥ 1 IU/l, testosterone ≥ 8 nmol/l, ejaculate volume ≥ 1.5 ml. Patients with genetic abnormalities or partners with female factors were excluded.

For the identified study population (n=2742), we used common andrologic features (somatic, semen and hormonal parameters, including the *FSHB* c.-211G>T (rs10835638) single nucleotide polymorphism) for subsequent analyses. Cluster analyses were performed for the entire study population and for two sub-cohorts, which were separated by total sperm count (TSC) thresholds: Cohort A (TSC ≥ 1 mill/ejac; n=2422) and Cohort B (TSC < 1 mill/ejac; n=320). For clustering, the *partitioning around medoids* method was employed, and the quality was evaluated by average silhouette width.

**Results:**

The applied cluster approach for the whole study population yielded two separate clusters, which showed significantly different distributions in bi-testicular volume, FSH and *FSHB* genotype. Cluster 1 contained all men homozygous for G (wildtype) in *FSHB* c.-211G>T (100%), while Cluster 2 contained most patients carrying a T allele (>96.6%). In the analyses of sub-cohorts A/B, two clusters each were formed too. Again, the strongest segregation markers between the respective clusters were bi-testicular volume, FSH and *FSHB* c.-211G>T.

**Conclusion:**

With this first unbiased approach for revealing putative subgroups within a heterogenous group of idiopathic infertile men, we did indeed identify distinct patient clusters. Surprisingly, across all diverse phenotypes of infertility, the strongest segregation markers were *FSHB* c.-211G>T, FSH, and bi-testicular volume. Further, Cohorts A and B were significantly separated by *FSHB* genotype (wildtype vs. T-allele carriers), which supports the notion of a contributing genetic factor. Consequently, *FSHB* genotyping should be implemented as diagnostic routine in patients with idiopathic infertility.

## Introduction

Around 30-75% of infertile men are diagnosed with idiopathic infertility, meaning that there are no obvious etiologic factors sufficient to explain the impaired fertility status (1–3). Idiopathic male infertility is a heterogenous disease, whereby sperm parameters can range from azoo- to normozoospermia, motility can be decreased and morphology can be reduced in these patients (4). While the term ‘unexplained infertile’ summarizes the cohort of men with semen parameters within regular range, and exclusion of female and etiologic factors (5). In this study we analysed men expressing all phenotypes of infertility from azoo- to normozoospermia. Since no causative factors have so far been identified for this group of idiopathic infertile men, causative treatment options cannot be offered, and these couples are usually referred to assisted reproductive techniques (ART). This puts the burden of treatment, e.g., hormonal stimulation, on the female partner and increases risks for progeny health (6, 7).

Several tools and parameters are currently featured in the workup for male infertility, such as semen analysis according to WHO standards, hormonal analysis, scrotal ultrasound and a thorough patient history looking for potential risk factors (1, 8, 9). However, even as early as two decades ago, Pierik and colleagues argued that more thorough diagnostic evaluations should be conducted to putatively identify subgroups which can be used for evidence-based studies on etiology, diagnosis and eventually treatment (10).

New analysis tools such as machine learning and cluster analysis can provide a more in-depth approach to these emerging clinical questions; however, such techniques require large sets of well-curated data. Having access to a database (Androbase^®^) (11) with data from more than 40,000 patients, we performed an unbiased cluster approach comprising 37 features (e.g., somatic, semen and hormonal parameters) with the goal of identifying subgroups in a cohort of men with idiopathic infertility. Such an explorative analysis has the potential to uncover hitherto hidden patterns in data that might be difficult to spot for andrologists but obvious to computers.

## Patients and Methods

### Study Population

**Figure 1:**
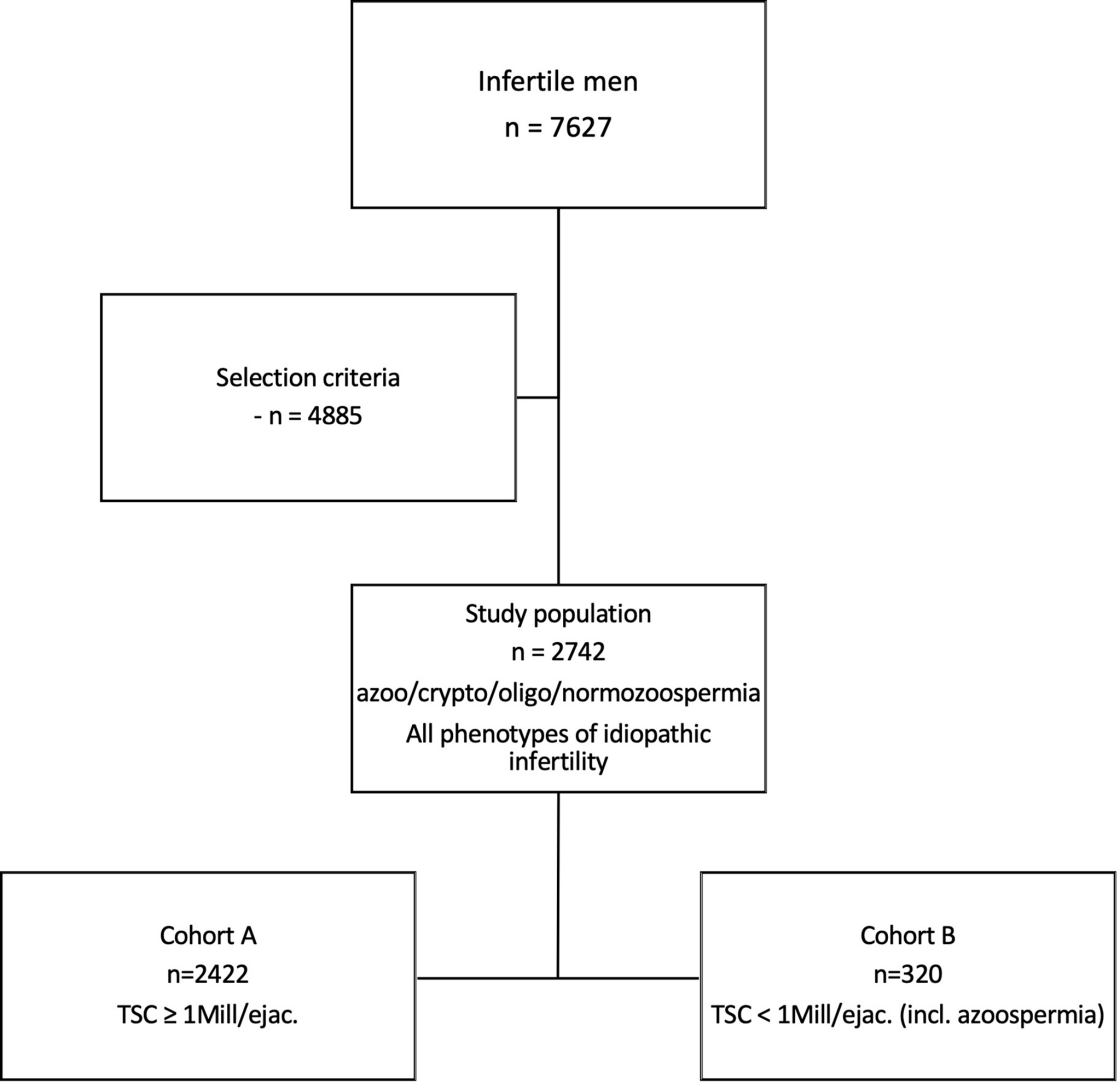
Selection of study population of idiopathic infertile men. In a retrospective query of our database with a ten-year perspective, 7627 infertile men were selected. After applying strict selection criteria, 2742 men with idiopathic infertility were identified. The entire study population includes all phenotypes of idiopathic male infertility, ranging from azoo- to normozoospermia. Cohort A, being a sub-cohort, includes n=2422 men with a total sperm count (TSC) ≥1 mill/ejac. Cohort B (n=320) comprises men with TSC <1 mill/ejac. in whom (m)TESE had been performed.

In a retrospective query of our Androbase^®^ database we searched for infertile men who had visited the Centre of Reproductive Medicine and Andrology (CeRA) within a 10-year period (2008-2018) for a fertility workup after seeking for children for more than 12 months of unprotected sexual intercourse; we identified 7627 patients (**Figure 1**).

To further select for men with idiopathic infertility, the following criteria were applied: FSH serum level ≥ 1 IU/l and testosterone level ≥ 8 nmol/l (to avoid hypogonadotropic hypogonadism as an etiologic factor). In the case of severe crypto- or azoospermia, we only included men for whom a (m)TESE procedure had been performed and for whom histological data as well as sperm retrieval rate (SRR) were available. To rule out other etiologic factors, we excluded patients with any anomaly in genetic testing (i.e., karyotype, AZF deletions, CFTR mutation and additional genes known to be associated with azoospermia or congenital hypogonadotropic hypogonadism). Further exclusion criteria included current or former oncological diseases, gonadotoxic treatments (including chemo- or radiotherapy), medication influencing the hypothalamic-pituitary-gonad axis and having a single testis only. Additionally, we also excluded any patients whose partner had any major female factors contributing to the infertility, like endometriosis, polycystic ovaries, tubal occlusion, or amenorrhea.

To avoid selecting for a (small) artificial idiopathic infertile cohort that lacks any other etiologic factors contributing to impaired fertility, we deliberately included men who in fact presented risk factors that, while potentially affecting fertility, were unlikely to result in severe impairment on their own. This was important, as we also aimed to elaborate whether these factors would affect the cluster formation. These parameters included smoking status, varicocele of the testis, testicular maldescent, microlithiasis testis, and colonization of the ejaculate with germ. In total, 37 common andrologic features were included in the cluster analysis (**Table 1**).

**Table 1:** Reproductive parameters of study population (entire study population and Cohort A/B).

	Study population	Parameters unavailable	Cohort A	Parameters unavailable	Cohort B	Parameters unavailable
	**N = 2742**		**N = 2422**		**N = 320**	
**Age (years)**	35.2 ± 5.9	0	35.4 ± 5.9	0	33.9 ± 5.8	0
	35 (18-63)		35 (18-63)		33 (21-60)	
**FSH (IU/l) [1-7]**	6.2 ± 5.6	0	5.1 ± 3.8	0	14 ± 9.6	0
	4.3 (1-60.8)		4 (1-42.5)		12.7 (1.5-60.8)	
**n FSH 1-7 IU/l (%)**	75,31%		81,01%		32,19%	
**n FSH > 7 IU/l (%)**	24,69%		18,99%		67,81%	
**LH (IU/l) [2-10]**	3.6 ± 2	0	3.4 ± 1.8	0	5.2 ± 2.8	0
	3.2 (0.6-26.2)		3.1 (0.6-26.2)		4.6 (0.9-16.5)	
**Testosterone (nmol/l) [≥ 12]**	16.9 ± 6.5	1	17 ± 6.6	0	16 ± 5.5	1
	15.7 (8-101.9)		15.8 (8-101.9)		14.9 (8-34.8)	
**Bi-testic. volume (ml)**	40.1 ± 14.2	1	41.3 ± 14.1	0	31.3 ± 12.2	1
	38 (8-119)		40 (16-119)		29 (8-89)	
**Ejaculate volume (ml)**	3.9 ± 1.7	1	3.9 ± 1.7	0	4.1 ± 1.9	1
	3.6 (1.5-20.4)		3.6 (1.5-14)		3.7 (1.5-20.4)	
**Total sperm count (Mill/ejac)**	103.1 ± 144.4	1	116.7 ± 148.4	0	0 ± 0.1	1
	51.7 (0-1654.9)		66.4 (1-1654.9)		0 (0-0.7)	
**n ≥ 39 Mill/ejac (%)**	55,22%		63,51%		0,00%	
**n < 39 Mill/ejac (%)**	44,75%		37,49%		100,00%	
						
						
**Sperm ab-motility (%)**	38.6 ± 18.4	0	43.5 ± 13.1	0	1.2 ± 5.2	0
	45 (0-81)		47 (0-81)		0 (0-51)	
**Sperm morphology (%)**	4.1 ± 2.9	467	4.1 ± 2.9	148	0	319
	4 (0-50)		4 (0-50)		0	
**FSHB c.-211G>T numbers (%)**						
**GG**	1950 (71.12%)	22 (0.80%)	1745 (72.05%)	1 (0.04%)	205 (64.06%)	21 (6.56%)
**GT**	705 (25.71%)		622 (25.68%)		83 (25.94%)	
**TT**	65 (2.37%)		54 (2.23%)		11 (3.44%)	
**Maldescensus testis in history**	388 (12.33%)		256 (10.57%)		82 (25.63%)	
**Varicocele testis**	628 (22.90%)		553 (22.83%)		75 (23.44%)	
**Nicotine abuse**	988 (36.03%)		874 (36.09%)		114 (35.63%)	
**Microlithiasis**	203 (7.40%)		177 (7.31%)		26 (8.13%)	
**Colonization of ejaculate**	454 (16.56%)		417 (17.22%)		37 (11.56%)	

Data are presented as mean ± SD and median (range). Reference range of hormones is indicated in square brackets. Columns named ‘parameters unavailable’ indicate the number of patients for which a certain parameter was not available or could not be obtained (i.e., morphology in azoospermic men) for analysis.

Applying these selection criteria, we eventually identified 2742 men with idiopathic male infertility (study population). This study group included all phenotypes of idiopathic male infertility, ranging from azoo- to normozoospermia.

Due to the nature of these diverse phenotypes, certain features like sperm morphology and motility are allocated unequally among the patients (i.e., morphology and motility cannot be assessed in azoopsermic men). Such parameters were therefore excluded when the entire study population was analysed. Yet, to include these valuable features for putative subgroup formation, we further divided the study population into two cohorts and performed respective cluster analyses; Cohort A (n=2422) included men with a total sperm count (TSC) ≥ 1 mill./ejac, and Cohort B (n=320) included men with TSC < 1 mill/ejac. in whom (m)TESE had been performed and respective data was present (**Figure 1**). Consequently, the parameters that were deliberately left out of the analysis for the entire study population were included and analysed in Cohort A for putative subgroup formation.

All patients provided written informed consent for the evaluation of their clinical data and genetic analysis of the donated DNA samples. The study was carried out in accordance with the protocols approved by the Ethics Committee of the Medical Faculty and the state medical board (Az. 2017-139-f-S).

### Clinical Workup and Laboratory Analyses

Routine clinical workup included physical examination, ultrasound of the testes (including volumetrics of the testes) and hormone and semen analysis according to WHO guidelines. The procedures on clinical workup, including ultrasound have been described previously (12, 13).

The hormonal analysis comprised measurements for FSH, luteinizing hormone (LH), total testosterone, free testosterone, prolactin, estradiol and sex hormone-binding globulin (SHBG) (14). Serum testosterone was measured by commercial ELISA kit (DRG Instruments GmbH, Marburg, Germany). Mean intra-assay coefficients of variation (CV) were below 2% and mean inter-assay CVs below 5%. Levels of free testosterone were calculated from levels of sex hormone-binding globulin (SHBG) and total serum testosterone according to the law of mass action, using 3.6 × 10^4^ L/mol as the association constant of testosterone with albumin and 1 × 10^9^ L/mol with SHBG (15). Serum concentrations of SHBG, LH, FSH, prolactin and estradiol were determined using highly specific time-resolved fluoro-immunoassays (Autodelfia, Freiburg, Germany).

Ejaculates were obtained by masturbation after sexual abstinence of 3-7 days, at two time points, and semen analysis was carried out according to the WHO guidelines (8, 16). Colonisation of the ejaculate with germs was evaluated *via* PCR (Chlamydia, Ureaplasma, Mycoplasma) or *via* culture dish. For the cluster analysis, this information was summarized into one categorical variable indicating whether germs were or were not present in the ejaculate.

### Genomic DNA Isolation

DNA was isolated from EDTA-preserved peripheral blood samples using the FlexiGene DNA Kit from Qiagen (QIAGEN, Düsseldorf, Germany) according to the manufacturer’s specifications. The concentration and quality of samples were measured with the spectrometer FLUOstar Omega (BMG Labtech).

### Genotyping

The SNPs rs10835638 (*FSHB* c.-211G>T) and rs1394205 (*FSHR* c.-29G>A) were analysed by TaqMan PCR assays and allelic discrimination (Genotyping Assay C:27829553_10 for rs10835638 and Genotyping Assay C:426553_10 for rs1394205) using the StepOnePlus detection system (Applied Biosystems) as described elsewhere (12).

### Data Preparation

For conducting the cluster analysis, patient data was pre-processed in R (17). First, each feature was assigned to an appropriate type, either numeric or categorical. For this purpose, any non-numerical entries included in numeric features were deleted and replaced with NA values.

Since cluster analysis is sensitive regarding outliers, i.e., observations with extreme values tend to dominate the results, all numeric data was checked for outliers. A classic rule for detecting outliers is to calculate the feature-wise z-score and exclude values above and below a specific threshold (18). For this study, a threshold of ± 3.5 was chosen. Z-scores were calculated separately for patients in Cohort A and B, since these groups are expected to show different distributions in many parameters. Values that were identified as outliers were then replaced by the 0.01 or 0.99 quantile of their respective distributions, a procedure known as Winsorization (19).

In the study data, a small number of patients had missing values in at least one feature (**Table 1**). Since most clustering algorithms cannot handle missing data, those values were imputed. Missing values in the data for this study were considered to be missing completely at random or missing at random, i.e., missing values are not dependent on any unobserved values (20). Such missing data can be imputed by multiple imputation methods without biasing the analysis results (21). For this study, the R library *missForest* was employed because it can handle study data with both numerical and categorical data (22). MissForest is a non-parametric method and thus, does not require the features to be normal distributed (22). *MissForest* was applied to Cohort A and B separately. The default parameter setting was used. **Table 1** was created using original data, i.e., without outlier correction or imputation of missing data.

### Clustering

For this study, cluster analysis was performed using R (17). As a first step, dissimilarity of observations, i.e., patients, was calculated. A metric that can be applied in the case of mixed-type data is the *Gower* metric (23), which assigns a dissimilarity score between 0 to 1 to each pair of observations, where 0 means that the observations are identical. The dissimilarity matrix based on the *Gower* metric was computed with the *daisy* function of the *cluster* library (24). It automatically standardizes observations prior to dissimilarity calculation such that each feature is distributed in the range of 0 to 1.

5In a second step, patients were clustered with the *partitioning around medoids* (PAM) method, which was originally introduced by Kaufman and Rousseeuw (1987) (25) and is available in R as the *pam* function of the *cluster* library (24). It computes a predefined number of groupings around medoids, i.e., an observation that has the lowest dissimilarity to all other observations in one grouping. For this study, PAM was employed for clusterings with 2 to 20 groupings.

To assess the validity of the clusterings, we calculated the average silhouette width. This parameter considers two important criteria for clustering: the distance to the closest cluster and the average dissimilarity of observations within one cluster. It ranges between -1 and 1, where 1 indicates a perfect clustering (26). To compare the groups that were created through clustering, we tested for each feature whether the distribution differed significantly between groups. For numeric features, the non-parametric Wilcox test was used, while the chi-square test was employed for categorical features. Since multiple tests were conducted, the alpha level was corrected to 0.05 divided by feature number. Clustering was represented in a plot created with the dimension reduction technic t-SNE (*Rtsne* library in R) (27, 28).

## Results

### Stratifying for Men With Idiopathic Infertility

#### Total Study Population: Azoo- to Normozoospermia

To identify subgroups within the heterogeneous group of idiopathic infertile men, we chose an unbiased cluster approach. For this, we retrospectively selected from our in-house database more than 7600 men displaying several etiologies of infertility. After applying strict selection criteria to rule out, e.g., genetic causes (see *Patients and Methods*), 2742 men with idiopathic infertility remained for subsequent analyses; they represent the entire study cohort. This cohort comprised all phenotypes of infertility, ranging from azoo- to normozoospermia.

#### Reproductive Parameters of Study Population

The median age of these patients was 35 years. Most men (75.3%) had serum FSH within the normal range (1-7 IU/l) with a median of 4.3 IU/l (range 1-61 IU/l) (**Table 1**). Median bi-testicular volume was 38 ml. Ejaculate analysis, according to WHO criteria, revealed a median total sperm count of 51.7 million sperm/ejaculate (range 0-1654.9 mill. sperm/ejac.). More than 28% (n=770) of these men were T-allele carriers in the *FSHB* c.-211G>T. More than one-third of the men were smokers, and 7.4% had a microlithiasis testis, diagnosed by scrotal ultrasound (**Table 1**).

#### Unbiased Cluster Analysis in the Entire Study Population Reveals Formation of Two Subgroups

**Figure 2:**
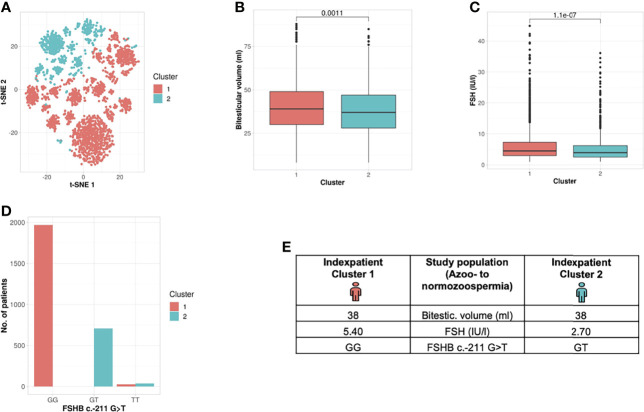
Cluster analysis entire study population: Identification of two clusters and three segregation parameters (azoo- to normozoospermia). **(A)** Distribution of patients in the clusters. Each dot represents one patient of the entire cohort (n=2742). The distances between the patients are presented in two-dimensional space. Silhouette width is 0.16. **(B–D)** The andrologic parameters bi-testicular volume, FSH and *FSHB* c.-211 are the significant segregation markers between the two clusters. The boxes show the first, second (i.e., median) and third quartiles of the respective parameters in Cluster 1 (comprising most individuals with GG) and Cluster 2 (comprising most individuals with a T allele). Outliers are shown as circles above the whiskers. The patients in the clusters differ significantly in regard to bi-testicular volume (p= 0.001*)*, FSH (p=1.1e-7), and *FSHB* c.-211 (p=2.2e-16). **(E)** Reproductive parameters of the index patient of Cluster 1 and 2. The index patient represents Cluster 1 or 2, respectively, with the parameters in which both clusters differ significantly. (The presented values do not necessarily match the median or mean of all parameters in the clusters.).

By clustering 27 andrological parameters in 2742 men with idiopathic infertility, two subgroups/clusters were observed (silhouette width: 0.16) (**Table 2** and **Figure 2**). Clustering was dominated by three parameters which differed significantly in both clusters, namely bi-testicular volume, FSH, and *FSHB* c.-211 (p= 0.0011/1.1e-07/< 2.2e-16). Together, these three parameters are the strongest segregation markers for the groups, resulting in the generation of two clusters with the smallest distance between members within one cluster. Notably, the clusters segregate closely along *FSHB* c.-211G>T: 100% of wildtype carriers (GG) could be allocated to Cluster 1, while 96.6% of men carrying a T allele were found in Cluster 2. The patients in the cluster of individuals with GG show a higher testicular volume and FSH than the patients in the cluster of individuals with T allele (**Figure 2**).

**Table 2:** Survey of all andrologic/histologic parameters that were included in the cluster analysis, and the respective p values.

		Analysis study population	Analysis Cohort A	Analysis Cohort B
**Silhoutte width**		0.158	0.119	0.103
**Clusters**		2	2	2
**Patients**		2742	2422	320
				
		**p value**	**p value**	**p value**
**Somatic**	**Age (years)**	0.660	0.540	0.500
	**Height (cm)**	0.002	0.130	0.002
	**Weight (kg)**	0.055	0.300	0.320
**Testes**	**Bi-testicular volume (ml)**	**0.001**	**6.00e-07**	**2.1e-07**
**Hormones**	**Free Testosterone (pmol/l)**	0.880	0.480	0.970
	**FSH (IU/l)**	**1.1e-07**	**1.8e-10**	**1.1e-11**
	**LH (IU/l)**	0.130	0.020	**2.2e-06**
	**Estradiol (pmol/l)**	0.210	0.280	0.100
	**Prolaktine (mU/l)**	0.520	0.670	0.018
	**SHBG (nmol/l)**	0.820	0.300	0.680
	**Testosterone (nmol/l)**	0.840	0.210	0.710
**Ejaculate**	**pH**	0.650	0.300	0.720
	**Ejaculate volume (ml)**	0.690	**2.3e-04**	0.830
	**Fructose (µmol/ejac.)**	0.790	**2.1e-04**	0.012
	**Glucosidase (mU/ejac.)**	0.790	0.100	0.270
	**Leukocytes (Mill/ml)**	0.520	0.060	0.480
	**Round Cells (Mill/ml)**	0.098	0.710	0.013
	**Zinc (µmol/ejac.)**	0.170	0.220	0.200
	**Abstinence (days)**	0.820	0.240	0.140
	**Total sperm count (Mill/ejac)**	0.043	0.010	–
	**Sperm morphology (%)**	0.760	0.380	–
	**Sperm ab-motility (%)**	–	**4.1e-04**	–
	**Sperm d-motility (%)**	–	**7.8e-05**	–
	**Sperm mid-piece defect (%)**	–	0.380	–
	**Sperm head defect (%)**	–	0.180	–
	**Sperm tail defect (%)**	–	0.920	–
	**Agglutination (%)**	–	0.520	–
	**Eosin (%)**	–	0.190	–
**TESE results**	**Sertoli Cell Only (%)**	–	–	**8e-23**
	**Sperm retrieval rate**	–	–	**1.1e-06**
**Other factors**	**Microlithiasis testis**	0.835	0.990	0.154
	**Germ Colonisation of ejaculate**	0.298	0.972	0.106
	**Maldescensus testis**	0.399	0.950	0.883
	**Varicocele testis**	0.089	0.014	0.009
	**Nicotine abuse**	0.890	0.566	0.959
	***FSHB* c.-211G>T**	**< 2.2e-16**	**< 2.2e-16**	**1.8e-15**
	***FSHR* c.-29G>A**	–	–	**2.3e-19**

The best-fit number of groupings as well as the silhouette width for this grouping is presented. All parameters that were considered in the analyses are listed. For each clinical parameter, the p value for a hypothesis of different distribution in the two clusters is shown. Significant p values are printed in bold font. Significance level was chosen as 0.05/# of features.

In an approach to translate these results into a clinical setting, we generated an index patient for each cluster representing the hallmarks of the respective cluster analyses. As such, the index patient has, on average, the shortest distance to all other patients of the cluster; the presented values do not necessarily match the median or mean of all parameters in the clusters. For the analysed study population, the index patient from Cluster 1 has a bi-testicular volume of 38 ml, a FSH measure of 5.4 IU/l, and he carries the wildtype (GG) in the *FSHB* c.-211G>T. In comparison, the index patient from Cluster 2 has a similar bi-testicular volume of 38 ml, an FSH value of 2.7 IU/l, and he carries a T allele in the *FSHB* c.-211G>T (**Figure 2**).

### Sub-Cohort Analyses

#### Cohort A: Oligo- to Normozoospermia

##### Reproductive Parameters of Cohort A

Depending on the underlying clinical pathology, certain parameters such as sperm counts in patients with azoospermia cannot be evaluated. To make sure these patients were included in putative cluster formation, we decided to separate the study population into sub-cohorts, namely Cohort A and B, using total sperm counts as a marker.

Cohort A included all men with a total sperm count ≥ 1 mill/ejac. The median FSH level in this cohort was 4.0 IU/l. Total sperm count, sperm motility and bi-testicular volume were slightly higher than in the entire study population (**Table 1**). The distribution of the genotypes of the *FSHB* c.-211G>T polymorphism was comparable to the entire study population, as was the number of smokers (36.1%) and the number of men with microlithiasis testis (7.31%) (**Table 1**).

##### Unbiased Cluster Analysis in Cohort A Identifies the Presence of Two Subgroups

**Figure 3:**
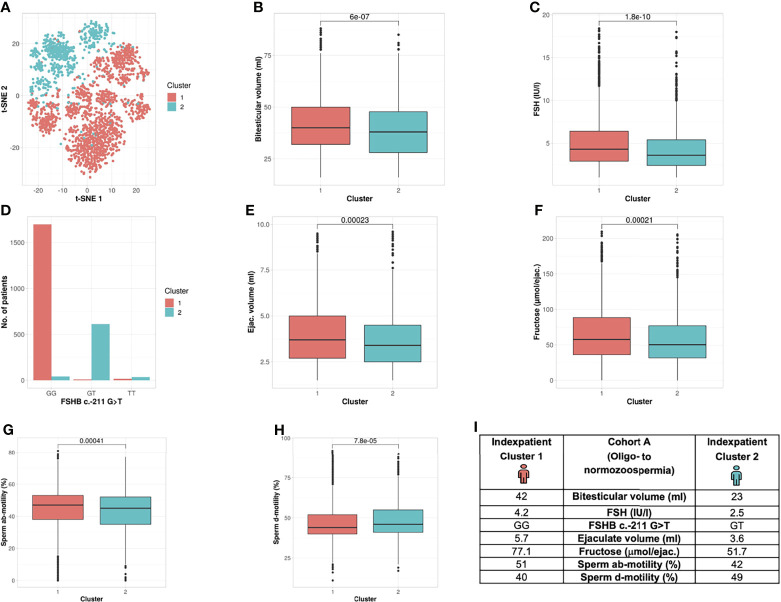
Cluster analysis Cohort A: Identification of two clusters and seven segregation parameters (oligo- to normozoospermia). **(A)** Distribution of patients in the clusters. Each dot represents one patient of the entire cohort (n=2422). The distances between the patients are presented in two-dimensional space. Silhouette width is 0.12. **(B–H)** The andrologic parameters bi-testicular volume, FSH and *FSHB* c.-211, ejaculate volume, fructose, ab- and d-motility are the significant segregation markers between the two clusters. The boxes show the first, second (i.e., median) and third quartiles of the respective parameters in Cluster 1 (comprising most individuals with GG) and Cluster 2 (comprising most individuals with a T allele). Outliers are shown as circles above the whiskers. The patients in the clusters differ significantly in regard to the mentioned seven andrologic parameters. Adjusted p values are located in the plots and range from (p < 2.2e^-16^ to 0.00023). **(I)** Reproductive parameters of the index patient of Cluster 1 and 2. The index patient represents Cluster 1 or 2, respectively, with the parameters in which both clusters differ significantly. (The presented values do not necessarily match the median or mean of all parameters in the clusters.).

Analysis of Cohort A revealed the formation of two clusters (silhouette width 0.12). Cluster formation was mainly driven by seven commonly analysed andrological parameters that were distributed significantly different between both clusters; the parameters were bi-testicular volume, FSH, *FSHB* c.-211G>T, ab-/d- motility, ejaculate volume and fructose (p <2.2 e ^-16^ – 0.00023) (**Table 2** and **Figure 3**). Again, the polymorphism *FSHB* c.-211 mirrored the segregation in the two clusters most accurately, allocating 97.6% of all wildtype carriers to Cluster 1 and 95.9% of all T-allele carriers to Cluster 2 within Cohort A (**Figure 3**). Here, the index patient of Cluster 1 differs in terms of the mentioned parameters from the representative patient of Cluster 2 (**Figure 3I**).

#### Cohort B: Azoo- to Cryptozoospermia

##### Reproductive Parameters of Cohort B

Cohort B comprised all men with a TSC ≤ 1 mill/ejaculate (n=320) and included only men in whom (m)TESE had been performed and information on the histological data as well as the sperm retrieval rate were present. These parameters, as well as *FSHR* -229G>A, were included in the analyses. Men in Cohort B showed a higher median FSH level (12.7 IU/l) and lower bi-testicular volume (29 ml) compared to those in Cohort A. Among this cohort, the relative amount of *FSHB* T-allele carriers was higher than in Cohort A and in the entire study population (**Table 1**).

##### Unbiased Cluster Analysis in Cohort B Reveals Formation of Two Subgroups

**Figure 4:**
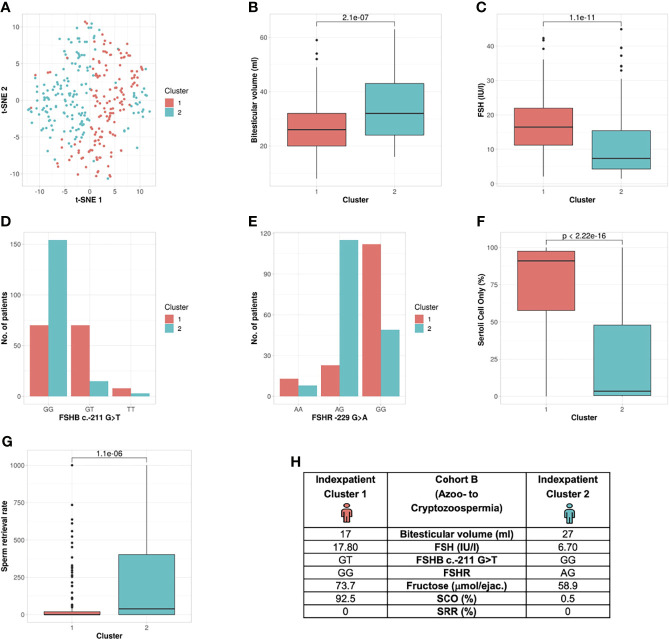
Cluster analysis Cohort B: Identification of two clusters and six segregation parameters (azoo- to cryptozoospermia). **(A)** Distribution of patients in the clusters. Each dot represents one patient of the entire cohort (n=320). The distances between the patients are presented in two-dimensional space. Silhouette width is 0.10. **(B–G)** The andrologic/histologic parameters bi-testicular volume, FSH and *FSHB* c.-211, FSHR, Sertoli cell only syndrome (SCO) and sperm retrieval rate (SRR) are the significant segregation markers between the two clusters. The boxes show the first, second (i.e., median) and third quartiles of the respective parameters in Cluster 1 (comprising most individuals with GG) and Cluster 2 (comprising most individuals with a T allele). Outliers are shown as circles above the whiskers. The patients in the clusters differ significantly in regards to the mentioned seven andrologic parameters. Adjusted p values are located in the plots and range from (p < 2.3e^-19^ to 2.2e^-6^). **(H)** Reproductive parameters of the index patient of Cluster 1 and 2. The index patient represents Cluster 1 or 2, respectively, with the parameters in which both clusters differ significantly. (The presented values do not necessarily match the median or mean of all parameters in the clusters.).

The unbiased cluster approach revealed two clusters (silhouette width: 0.10) significantly different in the following six parameters: bi-testicular volume, FSH, LH, *FSHB* c.-211G>T*, FSHR c.*-29G>A, proportion of Sertoli cells only (SCO) in the histologic sample, and sperm retrieval rate (SRR) (**Table 2** and **Figure 4**) (p value: 8e-23 – 0.0000011). In Cohort B, *FSHB* c.-211 showed a lower prediction rate for cluster membership of patients with crypto- and azoospermia: 81.3% of T-allele carriers were allocated to Cluster 1, and 68.8% of wildtype patients were assigned to Cluster 2. The index patients of both clusters differ in the mentioned parameters (**Figure 4H**).

### Impact of (other) Risk Factors on the Formation of Subgroups in All Three Study Groups (Study Population, Cohort A, Cohort B)

Frequently assessed parameters that are suspected to impact fertility, such as maldescensus testis, varicocele testis, nicotine abuse, germ colonization of the ejaculate and microlithiasis testis, were widely distributed among the clusters. However, none of these features were significant either as segregation markers or for subgroup formation. Therefore, in our analyses in idiopathic infertile men, these features did not have a significant impact on the formation of subgroups (entire study population, Cohort A or Cohort B) (data not shown).

Taken together, by our novel approach of cluster analyses, the previously heterogenous group of men with idiopathic infertility was able to be further subdivided. In all analyses, the bi-testicular volume, FSH and *FSHB* c.-211G>T were the analysed parameters that differed significantly between Clusters 1 and 2.

## Discussion

Idiopathic infertility is a common finding in the workup of an infertile couple, leaving these patients with an unsatisfactory diagnosis and no (causative) treatment besides ART. The diagnosis summarizes a heterogeneous group of infertile men; however, by applying an unbiased clustering approach, we aimed to identify a putative combination of parameters that cannot be readily identified by an attending physician and that may help categorize these patients into subgroups. Among the 37 common clinical and histologic parameters used for clustering, we found that bi-testicular volume, FSH and *FSHB* c.-211G>T were the three strongest segregation markers. The two separate clusters identified in our analyses were significantly different in these three features; this was the case for the analysis of the entire cohort (including all phenotypes of idiopathic infertility) as well as for the analyses of two sub-cohorts (A and B).

### Impact of Bi-Testicular Volume, FSH and FSHB on Cluster Formation

The three parameters bi-testicular volume, FSH, and FSHB certainly are related to one another: FSH is essential for the initiation and maintenance of spermatogenesis in humans. During the prenatal and prepubertal stages, FSH stimulates Sertoli cell proliferation and, by doing so, determines their final number and subsequently testicular size. In the adult stage, proliferation is ceased in mature Sertoli cells and FSH stimulates the proliferation of spermatogonia (29–31). Therefore, one can easily explain the combination of FSH and testicular volume being segregation parameters.

In big cohort studies, the single nucleotide polymorphism (SNP) *FSHB* c.-211G>T has been found to affect FSH serum levels, testicular size and sperm concentration (32–34). Also, given the functional impact of this SNP on the transcriptional rate of *FSHB* expression, the combination of FSHB, FSH and testicular volume as significant segregation markers is comprehensible (35).

Surprisingly, sperm parameters like total sperm count, motility and morphology played a minor role in cluster formation. Only in Cohort A (TSC ≥ 1 mill/ejac.) did the accessory glands play an important role as segregation markers: Here, the parameters ejaculate volume, fructose and ab- and d-motility differed significantly in patients between the clusters. The association between such parameters and the *FSHB* genotype has not been investigated so far and might be worth further investigation.

In the full study population and in Cohort A, we saw a trend for total sperm count (TSC) (p=0.043/0.010) as an important segregation marker. In the very severe phenotype of azoospermic men, sperm retrieval rate (SRR) can be an indicator of spermatogenic output. Indeed, SRR was a significant segregation marker (p < 0.0001) for Cohort B. This is in line with earlier studies in which SRR in (m)TESE was lower for patients carrying a T allele in *FSHB* c.-211 than for wildtype carriers (12). In a consecutive study, we showed that the T allele does not reduce the testicular Sertoli cell (SC) population, meaning that the spermatogenic output could putatively be increased pharmacologically (36).

We additionally used the cluster approach to evaluate the impact of multiple factors frequently collected in infertility workup that have a known impact with different effect size on fertility. To avoid creating an artificial study population, the following factors were included in our analyses: testicular maldescent, varicocele, smoking, germ colonization of the ejaculate and microlithiasis testis. None of the features were significantly distributed among the clusters in any analysis. In this big cohort approach, these factors are not helpful in forming subgroups that have, for example, more severe forms of infertility. For the individual patient, fertility can be impaired as a result of these factors (1, 8) and increase morbidity; however, in this big cohort approach, these factors do not contribute to subdividing or reducing the heterogenous group of infertile men.

### Including Genotyping of FSHB Into Clinical Workup of Idiopathic Infertile Men

While measuring FSH and assessing testicular volume are routine procedures in infertility workup (1, 9), the *FSHB* c.-211G>T single nucleotide polymorphism is not yet included in routine clinical diagnostics for infertile men. Given the proven impact of this SNP on a functional level as well as in big clinical cohort studies – and as we have shown its impressive impact as segregation marker – we suggest including the genotyping of *FSHB* into the routine andrological workup of men with idiopathic infertility.

The effect of this SNP as segregation marker is surprisingly tremendous in the entire cohort (including all phenotypes of impaired fertility). Due to the categorical nature of the SNP, the segregation is well comprehensible, in that all patients in the entire cohort carrying the wildtype (GG) were allocated to Cluster 1 and 96% of T-allele carriers to Cluster 2. This segregation effect is less pronounced in the azoo- and cryptozoospermic group (Cohort B), which can be explained by the decreasing impact of SNPs and the increasing impact of gross genetic anomalies (i.e. hitherto unknown mutations in relevant reproductive genes) in the more severe phenotypes (2, 37, 38).

The SNP outperforms strong semen parameters such as TSC, in that a clear allocation to one cluster was found for the genotype but was not found for TSC. We believe that the T allele in this polymorphism is a major contributing genetic factor in men diagnosed with idiopathic infertility: In most cases, these men have, in median, lower FSH levels due to an insufficient upregulation *via* feedback mechanism, which can potentially impair spermatogenesis. In our cohort, men carrying a T allele made up 28% of the individuals, meaning that in 28% of idiopathic infertile men, genotyping may identify a *secondary functional hypogonadism with isolated FSH deficiency*. In our view this term fits best to describe the situation of a functional hypogonadism. The isolated FSH deficiency is the consequence of the lack of upregulation of FSHB biosynthesis *via* feedback mechanism.

The number of idiopathic infertile men could be reduced by nearly one-third due to this etiologic/contributing factor. As such, we suggest that after thorough diagnostic fertility workup and the exclusion of other relevant etiologic factors (idiopathic infertility), genotyping of the *FSHB* c.-211G>T should be performed. Since the analysis itself resemble a commercially available PCR test system it can be implemented in the routine, the costs are modest and the benefit of identifying an etiologic factor outweighs the laboratory expenses. Moreover, it has been hypothesized – and should be analysed in prospective randomized trials – that these men could benefit from FSH treatment to increase spermatogenesis (7, 36, 39). This would even further support the subcategorization of diagnosed idiopathic infertile men using this polymorphism.

Interestingly, most of the now-identified segregation markers were determined by clinicians many years prior. However, in this study, using an unsupervised learning method allowed us to identify patterns inside the data (40) and to identify a combination of markers to create subgroups. A clustering solution creates groups of observations such that within a group the observations are as homogenous as possible, but between different groups they are as distinct as possible (41).

Examining the index patients, who resemble the centre of the respective clusters, the difference in testicular volume or FSH was low or equal in some analyses. Clinically, these patients seem alike; however, the genotype accounted for the differences, and the genotype was brought to light as a key segregation marker by the cluster analysis’s unsupervised learning approach. While artificial intelligence and its related concept of machine learning have been applied in reproductive medicine studies to improve success rate in ART *via* sperm and oocyte selection and ART prediction models (42), to our knowledge we are the first to apply this approach for diagnostic stratification.

This shows that big cohort studies are needed to identify the putative subgroups that can reveal significant differences in key parameters. For the first time we found that FSHB is a reliable segregation parameter, which was easy to identify because of its categorical nature. As such, in this case the machine learning approach emphasized the clinical impact/role of this polymorphism on subgroup formation.

### Limitations

Clustering results have the potential bias of patient and feature selection. If other selection criteria for this patient group were applied, the adapted feature set would result in a different clustering.

Categorical and numeric features contribute diversely to the calculation of patient dissimilarity. Potentially, categorical features can have a higher impact because patients are rated as completely different if they fall in different categories; for numeric features, the dissimilarity depends on the range of values. In this case, correlations with a smaller impact might be hidden by the dominant impact of *FSHB* c.- 211G>T.

## Conclusion

Using an unsupervised clustering approach based on several andrological parameters, we were able to subcategorize the heterogenous cohort of idiopathic infertile men into two subgroups. These two groups differ significantly in the main three parameters of *FSHB* c.-211G>T, FSH, and bi-testicular volume. The genetic parameter of *FSHB* c.-211G>T in combination with the established parameters FSH and testicular volume should attract more attention in future clinical workups of idiopathic infertile men.

Since the FSHB SNP was identified as a segregation marker, we suggest introducing diagnostic genotyping into the clinical routine for infertility workups of idiopathic infertile men, which will help identify men with secondary functional hypogonadism and isolated FSH deficiency. This may reduce the high number of diagnosed idiopathic infertile men by nearly one-third.

## Data Availability Statement

The data analyzed in this study contains data from patients attending the Centre of Reproductive Medicine and Andrology (CeRA), University Hospital Münster. Requests to access these datasets should be directed to Maria.Schubert@ukmuenster.de.

## Ethics Statement

The studies involving human participants were reviewed and approved by the Ethics Committee of the Medical Faculty and the state medical board (Ärztekammer Westfalen-Lippe) (Az. 2017-139-f-S). The patients/participants provided their written informed consent to participate in this study. Written informed consent was obtained from the individual(s) for the publication of any potentially identifiable images or data included in this article.

## Author Contributions

MS, JG, and HK designed the study, compiled the available literature, and wrote the manuscript. HK performed the bioinformatic and statistical analyses. SK was responsible for patient phenotyping. MS was responsible for the patient cohorts selection. JG was responsible for genotyping. MS, HK, and AS designed the figures. AS and SK gave valuable comments prior to the design of the study, during discussions of the outcome and on the manuscript. All authors contributed to the article and approved the submitted version.

## Funding

This work was supported by grants from German Research Foundation for the CRU 326 Male Germ Cells.

## Conflict of Interest

The authors declare that the research was conducted in the absence of any commercial or financial relationships that could be construed as a potential conflict of interest.

## Publisher’s Note

All claims expressed in this article are solely those of the authors and do not necessarily represent those of their affiliated organizations, or those of the publisher, the editors and the reviewers. Any product that may be evaluated in this article, or claim that may be made by its manufacturer, is not guaranteed or endorsed by the publisher.
